# Predictive value of baseline metabolic tumor volume for non-small-cell lung cancer patients treated with immune checkpoint inhibitors: A meta-analysis

**DOI:** 10.3389/fonc.2022.951557

**Published:** 2022-09-06

**Authors:** Ke Zhu, Danqian Su, Jianing Wang, Zhouen Cheng, Yiqiao Chin, Luyin Chen, Chingtin Chan, Rongcai Zhang, Tianyu Gao, Xiaosong Ben, Chunxia Jing

**Affiliations:** ^1^ Department of Public Health and Preventive Medicine, School of Medicine, Jinan University, Guangzhou, China; ^2^ International School, Jinan University, Guangzhou, China; ^3^ Department of Thoracic Surgery, Guangdong Provincial People’s Hospital, Guangdong Academy of Medical Sciences, Guangzhou, China; ^4^ Guangdong Key Laboratory of Environmental Pollution and Health, Jinan University, Guangzhou, China

**Keywords:** PET/CT (18)F-FDG, standardized uptake value, metabolic tumor volume, non-small-cell lung cancer, immune checkpoint inhibitor

## Abstract

**Background:**

Immune checkpoint inhibitors (ICIs) have emerged as a promising treatment option for advanced non-small-cell lung cancer (NSCLC) patients, highlighting the need for biomarkers to identify responders and predict the outcome of ICIs. The purpose of this study was to evaluate the predictive value of baseline standardized uptake value (SUV), metabolic tumor volume (MTV) and total lesion glycolysis (TLG) derived from 18F-FDG-PET/CT in advanced NSCLC patients receiving ICIs.

**Methods:**

PubMed and Web of Science databases were searched from January 1st, 2011 to July 18th, 2022, utilizing the search terms “non-small-cell lung cancer”, “PET/CT”, “standardized uptake value”, “metabolic tumor volume”, “ total lesion glycolysis”, and “immune checkpoint inhibitors”. Studies that analyzed the association between PET/CT parameters and objective response, immune-related adverse events (irAEs) and prognosis of NSCLC patients treated with ICIs were included. We extracted the hazard ratio (HR) with a 95% confidence interval (CI) for progression-free survival (PFS) and overall survival (OS). We performed a meta-analysis of HR using Review Manager v.5.4.1.

**Results:**

Sixteen studies were included for review and thirteen for meta-analysis covering 770 patients. As for objective response and irAEs after ICIs, more studies with consistent assessment methods are needed to determine their relationship with MTV. In the meta-analysis, low SUVmax corresponded to poor PFS with a pooled HR of 0.74 (95% CI, 0.57-0.96, P=0.02). And a high level of baseline MTV level was related to shorter PFS (HR=1.45, 95% CI, 1.11-1.89, P<0.01) and OS (HR, 2.72; 95% CI, 1.97-3.73, P<0.01) especially when the cut-off value was set between 50-100 cm^3^. SUVmean and TLG were not associated with the prognosis of NSCLC patients receiving ICIs.

**Conclusions:**

High level of baseline MTV corresponded to shorter PFS and OS, especially when the cut-off value was set between 50-100 cm^3^. MTV is a potential predictive value for the outcome of ICIs in NSCLC patients.

## 1 Introduction

Lung cancer is the most common cause of cancer-related deaths worldwide in 2020, accounting for 1.80 million deaths (1). Non-small-cell lung cancer (NSCLC), compromising 80-85% of the lung cancer cases (2), has raised significant public health concerns. NSCLC is mainly composed of squamous cell carcinoma and adenocarcinoma (3), and the 5-year survival rate is 25% (4).Clinically, more than 60% of NSCLC patients had locally progressed or metastatic diseases (stage III or IV) at the time of diagnosis, when the tumor can not be effectively treated by surgical treatment alone (5), and the median overall survival varies between 7.0 and 12.2 months (6).

For the treatment of advanced NSCLC, chemotherapy remains the primary conventional therapy. But the response rate of NSCLC patients to chemotherapy was only about 20% (7), and the adverse events such as vomiting and diarrhea had a significant impact on patients’ daily lives. The advent of immune checkpoint inhibitors (ICIs) targeting programmed cell death 1 (PD-1) or its ligand (PD-L1) has brought about a promising treatment option for the management of advanced NSCLC (8). A meta-analysis of 13 randomized controlled trials (RCTs) has proved that ICIs show better efficacy and result in fewer adverse events than chemotherapy as the treatment for advanced NSCLC (9). However, the benefits of ICIs remain limited to only 20% of advanced NSCLC patients (10). Thus it’s necessary to identify potential biomarkers to identify NSCLC patients who would benefit from ICIs treatment.

^18^F-fluorodeoxyglucose positron emission tomography/computed tomography (^18^F-FDG PET/CT) monitors the uptake of ^18^F-FDG of tumor cells. It is a convenient imaging modality for the staging, treatment guidance, and response predicting in NSCLC patients and is more practical and noninvasive than abdominal ultrasound and mediastinoscopy (11).

As metabolic parameters on PET/CT, SUV is associated with 18F-FDG uptake of the tumor; MTV combines the information of 18F-FDG uptake and tumor volume; TLG is the product of MTV and SUVmean, and is related to both tumor volume and tumor glycolytic activity. They reflect both tumor burden and aggressiveness (12). Takada et al. found that the accumulation of ^18^F-FDG as SUVmax and SUVmean in tumor cells was significantly associated with PD-L1 expression in NSCLC patients (13). In addition, MTV and TLG have been potential prognostic factors in NSCLC patients treated with surgery (14) and chemotherapy (15). Thus SUV, MTV and TLG are expected to evaluate the efficacy of ICIs in advanced NSCLC patients. However, relevant studies showed inconclusive results. Monaco et al. have demonstrated that NSCLC patients with MTV and TLG values lower than the median values had improved outcomes of ICIs compared to those with higher values (16). No significant relationship was found between MTV, TLG, and ICIs response in studies conducted by Yamaguchi et al. (17) and Castello et al. (18).

Thus, we conducted this meta-analysis to assess the predictive value of SUV, MTV and TLG for advanced NSCLC patients receiving ICIs.

## 2 Material and methods

### 2.1 Data search and study selection

From January 1^st^, 2011, to July 18^th^, 2022, We searched comprehensively English language publications from PubMed and Web of Science using the terms “non-small-cell lung cancer”, “PET/CT”, “Standardized uptake value”, “metabolic tumor volume”, “ total lesion glycolysis”, and “immune checkpoint inhibitors”. We extracted data from the full-text articles that met the following inclusion criteria: studies limited to NSCLC; ICIs administered alone for the patients; ^18^F-FDG PET/CT completed before ICIs initiation; studies reported objective response, immune-related adverse events (irAEs), survival data, including progression-free survival (PFS) or overall survival (OS); hazard ratio (HR) with 95% CI was provided for PFS or OS. Reviews, meeting abstracts, and editorial material were excluded. Two authors conducted the searches and screening independently. A consensus resolved any discrepancies.

### 2.2 Data extraction

Data were extracted from the publications independently by two reviewers (YC and CC), and the following information was recorded: first author’s name, year of the paper published, country, types of ICIs, median follow-up, number of patients, median age of patients, median values of MTV and TLG, HR and p-value for PFS and OS. The data were collected and organized in a standardized data extraction table for analysis. We also formed a table including median values of MTV or numbers of patients in different objective response groups and the related p-value to demonstrate the relationship between MTV and objective response. When there was uncertainty in the inclusion of data, a third researcher assisted with confirming the data.

### 2.3 Quality assessment

We used ROBINS-I (Risk Of Bias In Non-randomised Studies - of Interventions) to assess the quality of included articles from seven bias domains, including confounding bias, selection bias, bias due to classification of interventions, bias from intended interventions, bias due to missing data, bias in outcomes measure, and bias due to selection reporting result. We classified each article as low, moderate, or high risk according to detailed guidance from ROBINS-I (19).

### 2.4 Statistics analysis

We performed all statistical analyses using Review Manager v.5.4.1 and pooled the hazard ratio (HR) and its 95% confidence index (CI) of PFS and OS using the inverse variance method. An HR greater than 1 indicated worse survival for patients with high SUV, MTV or TLG, while an HR less than 1 indicated a better survival for patients with a high SUV, MTV or TLG. Chi-square test and *I^2^* statistics were used to detect heterogeneity between studies. *I^2^* values of more than 50% were considered high heterogeneity. If high heterogeneity was found between primary studies, a random effect model would be used for meta-analysis. Otherwise, a fixed effect model would be applied. P values less than 0.05 were considered statistically significant.

## 3 Results

### 3.1 Literature search

**Figure 1:**
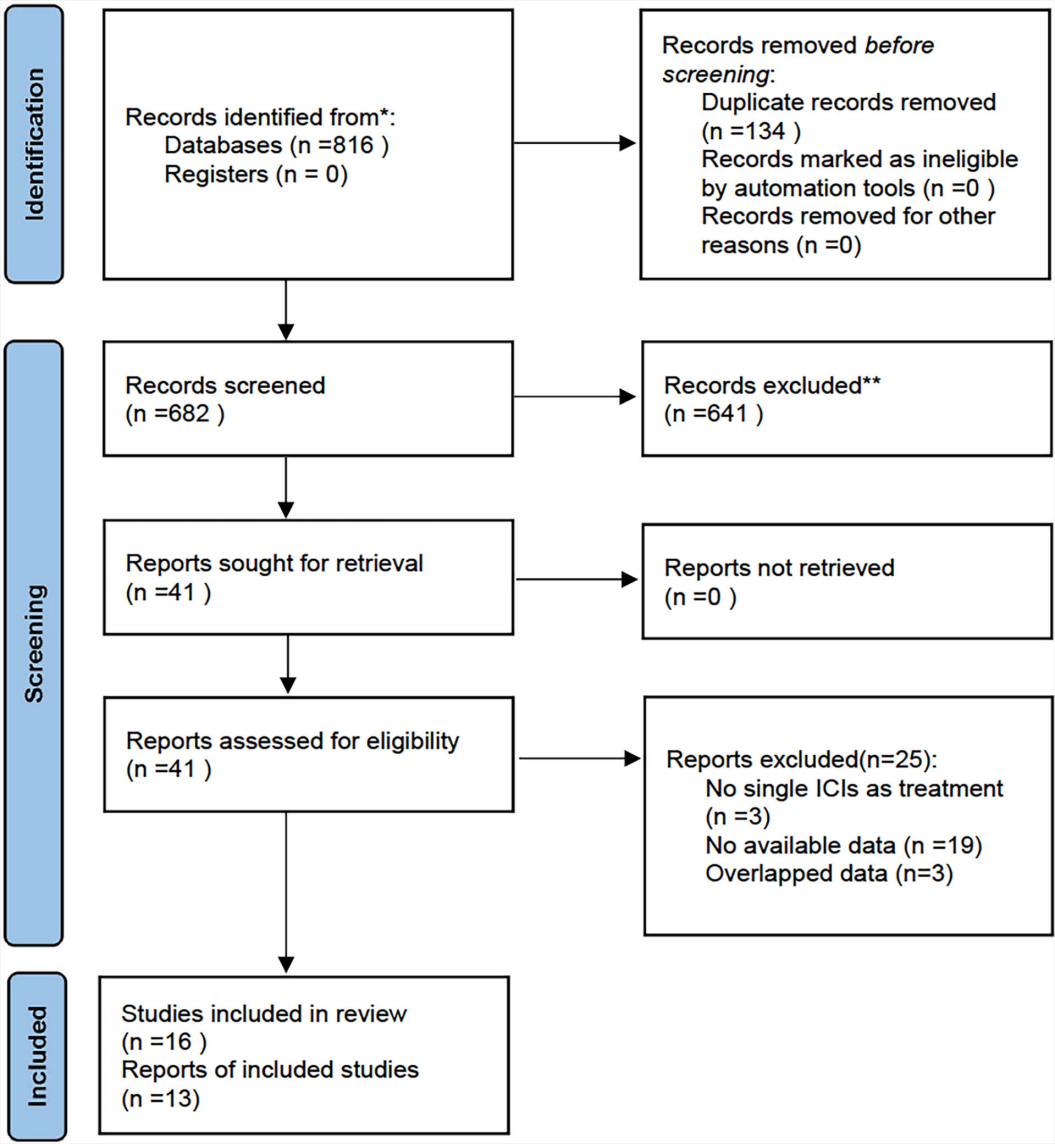
Flowchart diagram for the literature search.

Eight hundred and sixteen studies were retrieved from the systematic search of PubMed and Web of Science from January 1^st^, 2011, to July 18^th^, 2022. We excluded 134 duplicate studies and further screened the remaining 682 using titles and abstracts. 641 studies did not meet the inclusion criteria and thus were excluded. The full texts of the 41 potentially eligible studies were evaluated. Then 25 studies were excluded for the following reasons: not single ICIs as treatment (n=3), no available data (n = 19), and overlapped data (n=3). Ultimately, sixteen studies were included for review and thirteen studies assessing the predictive value of SUV, MTV and TLG in NSCLC patients receiving ICIs were included in this meta-analysis. **Figure 1** shows the flowchart diagram.

### 3.2 Characteristics of included studies

The thirteen articles, including 770 patients, were analyzed in this meta-analysis. Characteristics of the included studies are summarized in **Table 1**. Four studies were conducted in France (20, 22, 26, 27), followed by three in Italy (16, 18, 21) and three in Japan (16, 18, 21). We also identified a single study in the United States (19), Israel (24) and Belgium (28). Two studies were of a prospective design (18, 20). SUV, MTV and TLG were measured in four studies (18, 20, 25, 28) and MTV alone was measured in five studies (17, 19, 21, 22, 24).

**Table 1:** Characteristics and results of included studies.

Studies	Year	Country	Study design	Types of ICIs	Median follow-up	No. of patients	Median Age	Median values as cut-offs	Outcome			
									HR (95% CI) for PFS	p value	HR (95% CI) for OS	p value
Andraos et al. (19)	2022	USA	R	–	17.0 months	124	67	MTV: 87.8 (cut-offs: 88.0)	1.36 (0.91-2.01)	0.131	2.23 (1.35-3.69)	0.002
Castello et al. (18)	2021	Italy	P	Nivolumab/pembrolizumab/atezolizumab	12.4 months	50	73	SUVmax: 13.6	0.9 (0.5-1.8)	0.75	0.9 (0.4-2.0)	0.75
								SUVmean: 5.9	0.9 (1.0-1.7)	0.75	0.8 (0.4-1.9)	0.71
								MTV: 63.7	2.5 (1.2-4.8)	0.01	2.3 (1.0-5.3)	0.04
								TLG: 330.1	1.8 (0.9-3.6)	0.08	1.5 (0.7-3.6)	0.27
Chardin et al. (20)	2020	France	P	Nivolumab/pembrolizumab	12.3 months	79	64	SUVmax: 13.4	–	–	1.31 (0.63-2.75)	0.5
								SUVpeak: 9.7	–	–	1.15 (0.55-2.40)	0.7
								MTV:36.5	–	–	5.37 (2.17-13.3)	<0.0001
								TLG: 267.0	–	–	5.05 (2.05-12.5)	0.0001
Dall’Olio et al. (21)	2021	Italy	R	Pembrolizumab	20.3 months	34	66.6	MTV: 75.0	–	–	5.37 (1.72-16.77)	0.004
Eude et al. (22)	2022	France	R	Pembrolizumab	–	65	64.1	MTV: 188.3	–	–	1.314	0.012
Hashimoto et al. (23)	2020	Japan	R	Nivolumab/pembrolizumab	–	85	–	MTV: 17.8 (cut-offs: 5.0)	1.28 (0.97-1.73)	0.07	1.59 (1.09-2.45)	0.001
								TLG: 75.4 (cut-offs: 20.0)	1.21 (0.92-1.63)	0.16	1.47 (1.03-2.21)	0.03
Icht et al. (24)	2020	Israel	R	Nivolumab/pembrolizumab	–	58	65	MTV:12.95	1.1 (0.87-1.4)	0.4	1.2 (0.86-1.73)	0.26
Kitajima et al. (25)	2021	Japan	R	Nivolumab/pembrolizumab	36.8 months	40	69.1	SUVmax: 8.57	1.04 (0.49-2.18)	0.92	1.56 (0.67-3.69)	0.3
								MTV: 15.5	2.15 (1.03-4.73)	0.042	2.15 (1.03-4.73)	0.042
								TLG: 87.7	1.15 (0.55-2.42)	0.7	1.35 (0.59-3.13)	0.47
Monaco et al. (16)	2021	Italy	R	Nivolumab/pembrolizumab/atezolizumab	–	92	70	SUVmean: 4.9	0.365 (0.150-0.890)	0.027	0.261 (0.084-0.808)	0.02
								MTV: 94.9	1.139 (0.989-1.311)	0.07	1.221 (1.063-1.402)	0.005
Seban et al. (26)	2019	France	R	Nivolumab/pembrolizumab/atezolizumab	11.6 months	80	61.9	SUVmax: 12.8	0.8 (0.5-1.3)	0.35	0.9 (0.5-1.5)	–
								MTV: 75.0	1.0 (0.9-1.1)	0.25	3.1 (1.7-5.7)	0.0001
Seban et al. (27)	2020	France	R	Pembrolizumab	13.4 months	63	65	SUVmax: 18	0.6 (0.3-1.1)	0.11	0.6 (0.2-1.6)	0.31
								SUVmean: 10.1	0.5 (0.3-1.1)	0.04	0.8 (0.3-1.9)	0.56
								MTV: 84.0	2.1 (1.1-4.3)	0.02	3.1 (1.1-8.3)	0.03
Vekens et al. (28)	2021	Belgium	R	Pembrolizumab	20 months	30	67	SUVmax: 15.7	0.62 (0.39-0.98)	0.04	0.54 (0.29-1.01)	0.06
								SUVpeak: 10.2	1.43 (0.97-2.11)	0.07	1.71 (0.97-3.03)	0.06
								SUVmean: 6.06	1.76 (0.54-5.79)	0.35	1.51 (0.46-4.93)	0.5
								MTV: 123.9	1.01 (0.99-1.03)	0.25	1.01 (0.99-1.02)	0.29
								TLG: 802.6	0.99 (0.99-1.00)	0.29	0.99 (0.99-1.00)	0.42
Yamaguchi et al. (17)	2020	Japan	R	Pembrolizumab	346 days	48	69	MTV: 112.0 (cut-offs: 268.0)	1.49 (0.77-3.24)	0.32	1.57 (0.98-2.41)	0.04

ICIs, immune checkpoint inhibitors; SUV, standardized uptake value; MTV, metabolic tumor volume; TLG, total lesion glycolysisl; R, retrospective; P, prospective; PFS, progression-free survival; OS, overall survival.

Regarding types of ICIs, nine studies (17, 20–25, 27, 28) reported using PD-1 inhibitors, while three used PD-1 and PD-L1 inhibitors (16, 18, 26). Patients were divided into high or low SUV/MTV/TLG groups in each study based on the cut-off values, and their PFS/OS were analyzed. And eleven of the thirteen studies used median MTV/TLG as cut-off values (16, 18–22, 24–28). The left two used log-rank test (23) and receiver operating characteristic (ROC) curve analysis (17) to determine cut-off values, respectively.

### 3.3 Quality assessment

**Figure 2:**
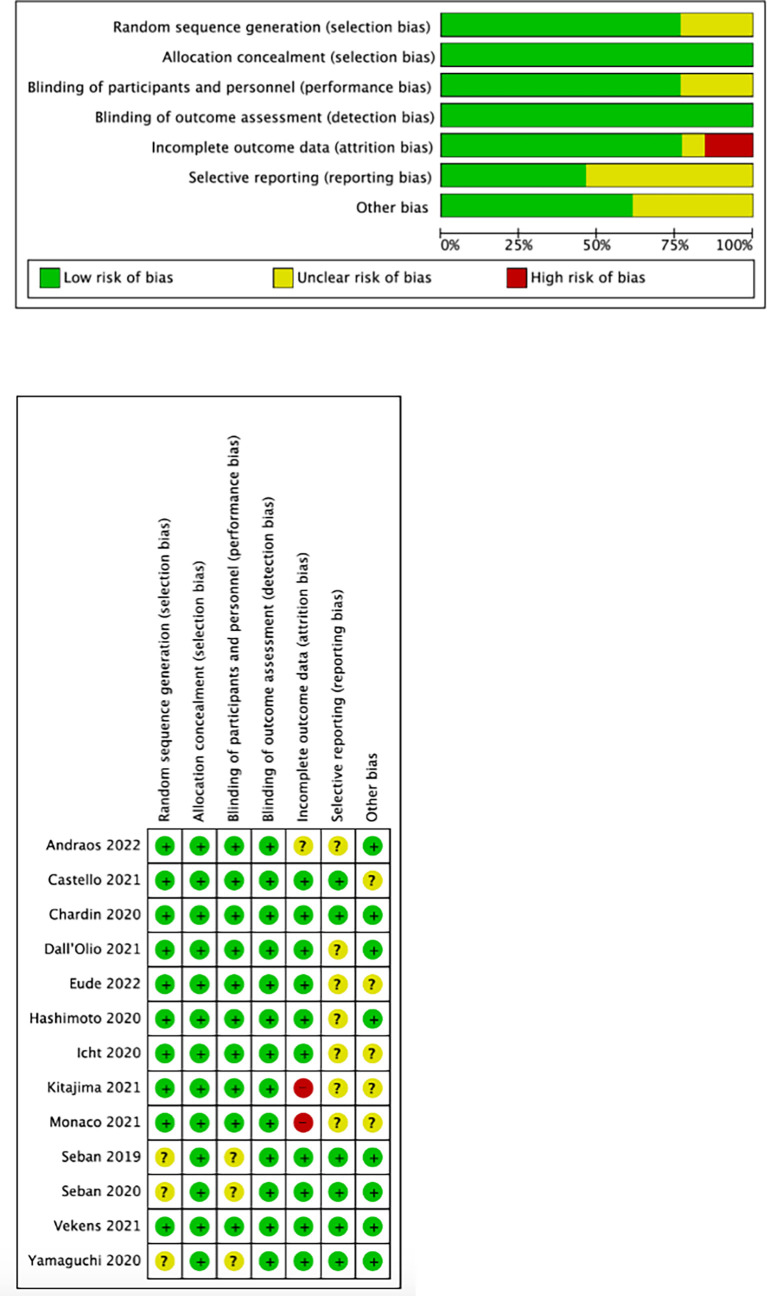
Results of quality assessment.

We used the Cochrane collaboration tool to assess the risk of bias in included studies. The risks of the selected studies are shown in **Figure 2**. As shown, the overall risk of bias was relatively low, and the overall quality met the requirements of the meta-analysis.

### 3.4 Outcomes of included studies

#### 3.4.1 PET/CT parameters and response assessment

Eight studies discussed whether PET/CT parameters including MTV, TLG, SUVmax, SUVmean, and SUVpeak, can predict the response of ICIs in different patients. All of them classified responses to ICIs as complete remission (CR), partial response (PR), stable disease (SD), and progression of disease (PD) based on the Response Evaluation Criteria In Solid Tumors (RECIST) 1.1. Four articles demonstrated that none of the PET/CT parameters significantly correlated with ICIs response (17, 23, 28, 29). However, the other four studies showed that NSCLC patients who achieved CR, PR, or SD after ICIs treatment had significantly lower median MTV values than those with PD (16, 26, 27, 30). The detailed data were shown in **Table 2**. In addition, Seban et al. found that SUVmean was significantly higher in patients who achieved long-term benefit (LTB, defined as CR, PR or SD maintained ±12 months) compared to those without LTB (27), while Polveri et al. concluded that TLG was significantly associated with progressive vs non-progressive disease status (30).

**Table 2:** MTV values and objective response (RECIST 1.1).

Authors	Published year	CR+PR+SD group		PD group		P value
		value	Number of patients	value	Number of patients	
*Median value of MTV*						
Ferrari et al. (29)	2021	203.0	15	–	13	0.387
Monaco et al. (16)	2021	77	61	160.2	31	**0.039**
Polvari et al. (30)	2020	57.4	27	124.4	30	**0.028**
Seban et al. (26)	2019	55.4	32	83.4	48	**0.04**
Seban et al. (27)	2020	59.4	17	90.5	46	0.05
Vekens et al. (28)	2021	192.8	23	119.8	7	0.17
*Number of patients in high/low MTV group*						
Hashimoto et al. (23)	2020	High MTV: 36 Low MTV: 17		High MTV: 18 Low MTV: 9		>0.99
Yamaguchi et al. (17)	2020	High MTV: 3 Low MTV: 20		High MTV: 7 Low MTV: 15		0.16

CR, complete response; PR, partial response; SD, stable disease; PD, progressive disease; MTV, metabolic tumor volume; Bold means statistically significant.

#### 3.4.2 PET/CT parameters and immune-related adverse events (irAEs)

Two studies discussed the relationship between PET/CT parameters and irAEs. In the analysis of Mu et al. (31), SUVmax and MTV were not correlated with irAEs, with the odds ratio of 0.95 (95%CI, 0.87-1.05, P=0.34) and 0.99 (95%CI, 0.98-1.00, P=0.27), respectively. However, Hashimoto et al. (23) reported that the frequency of irAE was significantly higher in patients with low values of SUVmax, MTV, and TLG than in those with high values, inconsistent with the result of Mu et al.

#### 3.4.3 PET/CT parameters and NSCLC survival

##### 3.4.3.1 SUVmax and NSCLC survival

**Figure 3:**
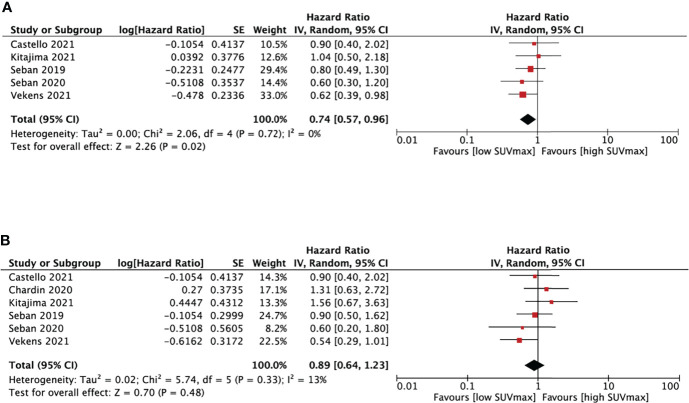
Forest plots of hazard ratios comparing progression free survival **(A)** or overall survival **(B)** of patients with high level versus low level max standardized uptake value treating with immune checkpoint inhibitors.

Six studies (18, 20, 25–28) analyzed the relationship between SUVmax and PFS/OS, as shown in **Figure 3**. The cut-off values of SUVmax ranged from 8.57 to 18 cm^3^. Five studies analyzing PFS showed a pooled HR of 0.74 (95% CI, 0.57-0.96, P=0.02). However, SUVmax was not significantly associated with OS (HR, 0.89; 95% CI, 0.64-1.23, P=0.48). There was no significant heterogeneity between studies in both PFS (I^2^ = 0%, P=0.72) and OS group (I^2^ = 13%, P=0.33).

##### 3.4.3.2 SUVmean and NSCLC survival

**Figure 4:**
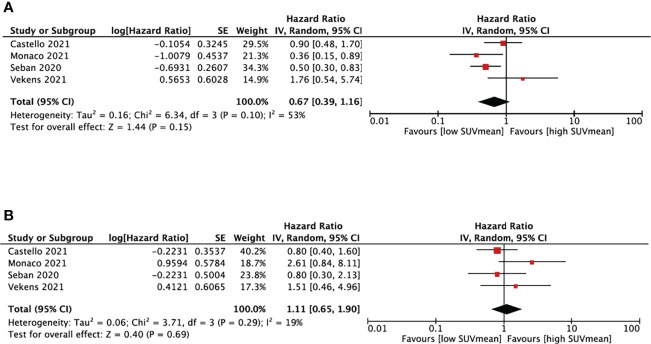
Forest plots of hazard ratios comparing progression free survival **(A)** or overall survival **(B)** of patients with high level versus low level mean standardized uptake value treating with immune checkpoint inhibitors.

We performed SUVmean and survival analysis based on four studies (16, 18, 27, 28) with cut-off values between 4.9 and 10.1cm^3^ (**Figure 4**). SUVmean was not associated with either PFS (HR, 0.67; 95% CI, 0.39-1.16, P=0.15) or OS (HR, 1.11; 95% CI, 0.65-1.19, P=0.69). The heterogeneity test didn’t show significant heterogeneity in PFS (I^2 =^ 53%, P=0.1) and OS group (I^2^ = 19%, P=0.29).

##### 3.4.3.3 MTV and NSCLC survival

**Figure 5:**
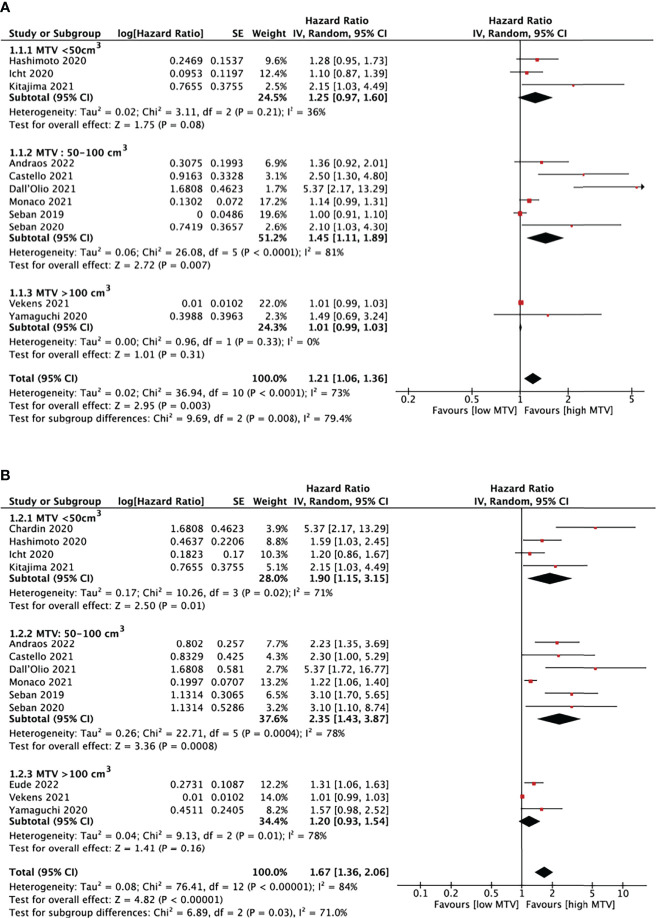
Forest plots of hazard ratios comparing progression free survival **(A)** or overall survival **(B)** of patients with high level versus low level metabolic tumor volume treating with immune checkpoint inhibitors.

Thirteen studies (16–28) analyzed the relationship between MTV and PFS/OS, as shown in **Figure 5**. The cut-off values of MTV ranged from 5.0 to 268.0cm^3^, so we performed a subgroup analysis based on the cut-off values, dividing them into three groups: MTV < 50cm^3^, MTV between 50-100cm^3^, and MTV >100cm^3^.

In eleven studies analyzing PFS, a pooled HR of 1.21 (95% CI, 1.06-1.36, P<0.01) was shown. There was statistically significant heterogeneity between studies, with an I^2^ of 79.4% (P<0.01). It is also demonstrated that patients with higher MTV would have shorter PFS (HR=1.45, 95% CI, 1.11-1.89, P<0.01) when the cut-off values was set at 50-100cm^3^. There was no evidence of a significant association between MTV and PFS in the other two subgroups.

OS was analyzed in thirteen MTV studies. The pooled HR was 1.67 (95% CI, 1.36-2.06, P<0.01) with statistically significant heterogeneity between studies (I^2^ = 84%, P<0.01). High MTV was significantly associated with poor OS, with an HR of 1.90 (95% CI, 1.15-3.15, P=0.01) and 2.35 (95% CI, 1.43-3.87, P<0.01) when the cut-off value was set below 50cm^3^ and 50-100cm^3^, respectively. The left subgroup showed no evidence of significant association.

##### 3.4.3.4 TLG and NSCLC survival

**Figure 6:**
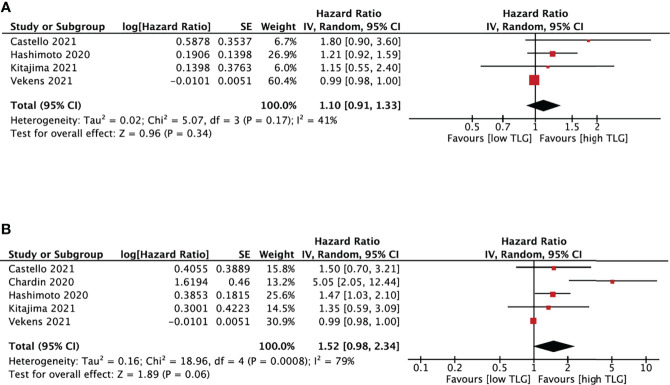
Forest plots of hazard ratios comparing progression free survival **(A)** or overall survival **(B)** of patients with high level versus low level total lesion glycolysis treating with immune checkpoint inhibitors.

TLG and survival analysis was performed based on five studies (18, 20, 23, 25, 28) with cut-off values between 20 and 802.6 (**Figure 6**). TLG was not associated with either PFS (HR, 1.10; 95% CI, 0.91-1.33, P=0.34) or OS (HR, 1.52; 95% CI, 0.98-2.34, P=0.06). The heterogeneity test showed high heterogeneity in OS (I^2 =^ 79%, P<0.01) and no significant results in PFS (I^2^ = 41%, P=0.17).

## 4 Discussion

This study evaluated the predictive values of PET/CT parameters including SUVmax, SUVmean, MTV and TLG in NSCLC patients receiving ICIs. The cut-off values categorized patients into high or low-level parameter groups in the included studies.

Firstly, we analyzed the relationship between PET/CT parameters and the objective response of ICIs. Eight studies assessed the objective response based on RECIST 1.1. Four studies showed NSCLC patients who achieved CR, PR, or SD after ICIs treatment had significantly lower median MTV values than those with PD (16, 26, 27, 30), while four demonstrated no significant correlation (17, 23, 28, 29). More studies with consistent response assessments are needed to determine whether MTV is associated with the objective response of ICIs. SUVmean (27) and TLG (30) were also said to have a significant relationship with disease status in a single study, respectively.

ICIs may alter the physiological homeostasis of the immune response, thus leading to the development of irAEs. Two studies discussed the relationship between PET/CT parameters and irAEs (23, 31). However, no consistent results could be yet concluded.

We also discussed whether PET/CT parameters could predict NSCLC survival by PFS and OS after ICIs. We found that lower SUVmax corresponded to shorter PFS. Lopci et al. found a positive association between SUVmax and CD8-tumor infiltrating lymphocytes and PD-1 expression (32). SUVmax were also independent predictors of PD-L1 positivity by Takada et al. (13). However, the predictive role of baseline SUVmax is still under discussion since only one of the five included studies about SUVmax showed significant results.

In terms of MTV, we found that a high baseline MTV level was significantly associated with shorter PFS and OS than a low MTV level for patients treated with ICIs.

MTV refers to the metabolically active volume of tumors segmented using FDG PET (33), reflecting tumor burden and the metabolic status. Regarding tumor burden, Kim et al. concluded that larger-size tumors are more immunosuppressive than smaller-size tumors, which negatively affects the immune responses induced by immunotherapy (34).The experiments in mice also verified that PD-L1 blocker is less effective in mice bearing larger lung squamous cell tumors (35). On the cell level, Wang et al. analyzed one hundred twenty-two NSCLC tumor specimens by immunohistochemistry and found a significantly positive correlation between MTV and CD163-TAM, Foxp3-Tregs (36). CD163-TAMs were tumor-promoting M2 macrophages (37), and Foxp3-Tregs were a kind of immune regulatory cells (35), both of which are immunosuppressive cells. Therefore, we hypothesize that patients with a higher MTV would have a worse prognosis when treated with ICIs than those with a lower MTV, since a higher MTV would result in a more immunosuppressive tumor microenvironment.

In respective of tumor glycolysis, a higher MTV indicates a larger metabolically active volume of glucose uptake by the tumor (38). Different from normal cells, tumor cells can uptake a large amount of glucose at a rapid rate, consuming most of nutrients from the surrounding environment, and metabolizing glucose into lactic acid (Warburg effect) (39). Tumors with higher MTV would have worse response to ICIs by affecting T cells responsiveness by the following possible ways.

Firstly, in tumor microenvironment (TME), tumor cells and T cells compete for glucose as their primary energy source (40). Tumors with higher MTV would consume more glucose and lead to glucose deprivation of T cells, decreasing T cells’ ability to produce effector cytokines like interferon gamma (IFN-γ), which has impact on the function of tumor infiltrating CD8+ T cells (41).

In contrast, Harley et al. found that melanoma tumors with less glycolysis would provide more glucose for infiltrating T cells and are associated with increased antigen presentation and better response to anti-PD-1 ICIs (42). Secondly, the accumulation of lactate in the TME will inhibit CD8+T cell proliferation and activation by preventing lactic acid export from CD8+T cell (43) or inhibiting CD25 expression, a T cells activation marker (44). More studies are still needed to explain why MTV could predict the outcome of immunotherapy in patients with NSCLC.

Since the cut-off values of MTV ranged from 5.0 to 268.0 cm^3^ in different studies, we also did a subgroup analysis to determine the impact of cut-off values on outcome assessment. Our result showed that a high baseline MTV level was significantly associated with shorter PFS when setting the cut-off values of MTV below 50cm^3^ and shorter OS in the groups with cut-off values lower than 50 cm^3^ or between 50cm^3^ and 100 cm^3^.

The baseline MTV level didn’t show any predictive value when the cut-off values were more than 100 cm^3^. Thus further studies with a larger sample size should focus on cut-off values of MTV between 50 and 100 cm^3^ and try to figure out a more precise cut-off value to improve the efficacy of MTV prediction on response assessment to ICIs in NSCLC patients.

Although SUVmean and TLG were potential prognostic markers of NSCLC (45), our pooled results showed that they were not significantly associated with PFS and OS in NSCLC patients receiving ICIs.

In addition to NSCLC, PET/CT parameters also played potential predictive roles in other cancers treated with ICIs, supporting our findings. Zhang et al. reported that total SUVmax ≥12.5 was associated with worse PFS in head and neck squamous cell carcinoma (46). And according to a systematic review and meta-analysis of metastatic melenoma (47), MTV and TLG were promising predictors of OS for metastatic melanoma patients who received ICIs.

^18^F-FDG PET/CT is a convenient and noninvasive imaging modality, and SUVmax and MTV are easily obtained. Since our study proved that SUVmax and MTV have the potential predictive value for ICIs in NSCLC patients, further studies are needed to define the role of SUVmax and MTV in providing individualized treatments for advanced NSCLC patients. Early identification of NSCLC patients for ICIs can improve the efficacy of ICIs in responders and avoid the side effects and high costs of ICIs in non-responders, allowing them to initiate other treatments timely.

Our study also has several limitations. Firstly, majority of the included studies are retrospective studies. Potential selection bias may exist and impact the reliability of this meta-analysis. Secondly, the methods of PET/CT were not consistent between different studies. A golden method should be defined to ensure the homogeneity of studies. Thirdly, cut-off values of SUV, MTV and TLG ranged widely and were determined by different methods, including median values, log-rank test and ROC curve analysis. Thus the pooled results may show some risk of bias.

In conclusion, our study showed that high baseline MTV levels correspond to shorter PFS and OS compared with low baseline MTV levels especially when the cut-off value was set between 50-100 cm^3^. MTV is a potential predictor of ICI outcomes in NSCLC patients.

## Data availability statement

The original contributions presented in the study are included in the article/supplementary material. Further inquiries can be directed to the corresponding author.

## Author contributions

CJ, TG and KZ conceived and designed research. Data collection was performed by DS, JW and ZC. Data extraction was performed by YC and CC and verified by KZ. Statistical analysis was performed by LC, RZ and KZ. KZ, DS, JW, ZC, YC, LC, CC, XB and CJ participated in drafting article. All authors gave final approval to the version submitted.

## Funding

This work was funded by the Guangdong Pharmaceutical Association Scientific Research Fund (No: 2021ZX03).

## Conflict of interest

The authors declare that the research was conducted in the absence of any commercial or financial relationships that could be construed as a potential conflict of interest.

## Publisher’s note

All claims expressed in this article are solely those of the authors and do not necessarily represent those of their affiliated organizations, or those of the publisher, the editors and the reviewers. Any product that may be evaluated in this article, or claim that may be made by its manufacturer, is not guaranteed or endorsed by the publisher.

## References

[1] World Health Organization . Cancer (2020). Available at: https://www.who.int/news-room/fact-sheets/detail/cancer (Accessed December 13, 2021).

[2] Salehi-Rad R Li R Paul MK Dubinett SM Liu B . The biology of lung cancer: Development of more effective methods for prevention, diagnosis, and treatment. Clin Chest (2020) 41(1):25–38. doi: 10.1016/j.ccm.2019.10.003 32008627

[3] Wang BY Huang JY Chen HC Lin CH Lin SH Hung WH . The comparison between adenocarcinoma and squamous cell carcinoma in lung cancer patients. J Cancer Res Clin Oncol (2020) 146(1):43–52. doi: 10.1007/s00432-019-03079-8 31705294 PMC11804334

[4] Cancer. lung cancer - non-small cell: Statistics (2021). Available at: https://www.cancer.net/cancer-types/lung-cancer-non-small-cell/statistics (Accessed December 13, 2021).

[5] Osmani L Askin F Gabrielson E Li QK . Current WHO guidelines and the critical role of immunohistochemical markers in the subclassification of non-small cell lung carcinoma (NSCLC): Moving from targeted therapy to immunotherapy. Semin Cancer Biol (2018) 52:103–9. doi: 10.1016/j.semcancer.2017.11.019 PMC5970946 29183778

[6] Schad F Thronicke A Steele ML Merkle A Matthes B Grah C . Overall survival of stage IV non-small cell lung cancer patients treated with viscum album l. @ in addition to chemotherapy, a real-world observational multicenter analysis. PloS One (2018) 13(8):e0203058. doi: 10.1371/journal.pone.0203058 30148853 PMC6110500

[7] Scheff RJ Schneider BJ . Non-small-cell lung cancer: Treatment of late stage disease: Chemotherapeutics and new frontiers. Semin Intervent Radiol (2013) 30(2):191–8. doi: 10.1055/s-0033-1342961 PMC3710022 24436536

[8] Kim JH Kim HS Kim BJ . Prognostic value of KRAS mutation in advanced non-small-cell lung cancer treated with immune checkpoint inhibitors: A meta-analysis and review. Oncotarget (2017) 8(29):48248–52. doi: 10.18632/oncotarget.17594 PMC5564642 28525386

[9] Yu DP Cheng X Liu ZD Xu SF . Comparative beneficiary effects of immunotherapy against chemotherapy in patients with advanced NSCLC: Meta-analysis and systematic review. Oncol Lett (2017) 14(2):1568–80. doi: 10.3892/ol.2017.6274 PMC5529907 28789381

[10] Song P Yang D Wang H Cui X Si X Zhang X . Relationship between the efficacy of immunotherapy and characteristics of specific tumor mutation genes in non-small cell lung cancer patients. Thorac Cancer (2020) 11(6):1647–54. doi: 10.1111/1759-7714.13447 PMC7262886 32342665

[11] Hochhegger B Alves GR Irion KL Fritscher CC Fritscher LG Concatto NH . PET/CT imaging in lung cancer: Indications and findings. J Bras Pneumol (2015) 41(3):264–74. doi: 10.1590/S1806-37132015000004479 PMC4541763 26176525

[12] Lim R Eaton A Lee NY Setton J Ohri N Rao S . 18F-FDG PET/CT metabolic tumor volume and total lesion glycolysis predict outcome in oropharyngeal squamous cell carcinoma. J Nucl Med (2012) 53(10):1506–13. doi: 10.2967/jnumed.111.101402 22895812

[13] Takada K Toyokawa G Okamoto T Baba S Kozuma Y Matsubara T . Metabolic characteristics of programmed cell death-ligand 1-expressing lung cancer on 18 f-fluorodeoxyglucose positron emission tomography/computed tomography. Cancer Med (2017) 6(11):2552–61. doi: 10.1002/cam4.1215 PMC5673920 28980429

[14] Liu J Dong M Sun X Li W Xing L Yu J . Prognostic value of 18F-FDG PET/CT in surgical non-small cell lung cancer: A meta-analysis. PloS One (2016) 11(1):e0146195. doi: 10.1371/journal.pone.0146195 26727114 PMC4699812

[15] Li X Wang D Yu L . Prognostic and predictive values of metabolic parameters of 18F-FDG PET/CT in patients with non-small cell lung cancer treated with chemotherapy. Mol Imaging (2019) 18:1536012119846025. doi: 10.1177/1536012119846025 31144578 PMC6545646

[16] Monaco L Gemelli M Gotuzzo I Bauckneht M Crivellaro C Genova C . Metabolic parameters as biomarkers of response to immunotherapy and prognosis in non-small cell lung cancer (NSCLC): A real world experience. Cancers (Basel) (2021) 13(7):1634. doi: 10.3390/cancers13071634 33915801 PMC8037395

[17] Yamaguchi O Kaira K Hashimoto K Mouri A Shiono A Miura Y . Tumor metabolic volume by 18F-FDG-PET as a prognostic predictor of first-line pembrolizumab for NSCLC patients with PD-L1 ≥ 50. Sci Rep (2020) 10(1):14990. doi: 10.1038/s41598-020-71735-y 32929123 PMC7490347

[18] Castello A Rossi S Toschi L Lopci E . Impact of antibiotic therapy and metabolic parameters in non-small cell lung cancer patients receiving checkpoint inhibitors. J Clin Med (2021) 10(6):1251. doi: 10.3390/jcm10061251 33803006 PMC8002619

[19] Andraos TY Halmos B Cheng H Huntzinger C Shirvani SM Ohri N . Disease Burden on PET Predicts Outcomes for Advanced NSCLC Patients Treated with First-Line Immunotherapy. Clin Lung Cancer (2022) 23(4):291–9. doi: 10.1016/j.cllc.2022.02.003 35382980

[20] Chardin D Paquet M Schiappa R Darcourt J Bailleux C Poudenx M . Baseline metabolic tumor volume as a strong predictive and prognostic biomarker in patients with non-small cell lung cancer treated with PD1 inhibitors: A prospective study. J Immunother Cancer (2020) 8(2):e000645. doi: 10.1136/jitc-2020-000645 32709713 PMC7380842

[21] Dall'Olio FG Calabrò D Conci N Argalia G Marchese PV Fabbri F . Baseline total metabolic tumour volume on 2-deoxy-2-[18F]fluoro-d-glucose positron emission tomography-computed tomography as a promising biomarker in patients with advanced non-small cell lung cancer treated with first-line pembrolizumab. Eur J Cancer (2021) 150:99–107. doi: 10.1016/j.ejca.2021.03.020 33892411

[22] Eude F Guisier F Salaün M Thiberville L Pressat-Laffouilhere T Vera P . Prognostic value of total tumour volume, adding necrosis to metabolic tumour volume, in advanced or metastatic non-small cell lung cancer treated with first-line pembrolizumab. Ann Nucl Med (2022) 36(3):224–34. doi: 10.1007/s12149-021-01694-5 35060071

[23] Hashimoto K Kaira K Yamaguchi O Mouri A Shiono A Miura Y . Potential of FDG-PET as prognostic significance after anti-PD-1 antibody against patients with previously treated non-small cell lung cancer. J Clin Med (2020) 9(3):725. doi: 10.3390/jcm9030725 PMC7141299 32156047

[24] Icht O Domachevsky L Groshar D Dudnik E Rotem O Allen AM . Lower tumor volume is associated with increased benefit from immune checkpoint inhibitors in patients with advanced non-small-cell lung cancer. Asia Pac J Clin Oncol (2021) 17(2):e125–31. doi: 10.1111/ajco.13360 32762128

[25] Kitajima K Kawanaka Y Komoto H Minami T Yokoi T Kuribayashi K . The utility of 68F-FDG PET/CT for evaluation of tumor response to immune checkpoint inhibitor therapy and prognosis prediction in patients with non-small-cell lung cancer. Hell J Nucl Med (2021) 24(3):186–98. doi: 10.1967/s002449912402 34901959

[26] Seban RD Mezquita L Berenbaum A Dercle L Botticella A Le Pechoux C . Baseline metabolic tumor burden on FDG PET/CT scans predicts outcome in advanced NSCLC patients treated with immune checkpoint inhibitors. Eur J Nucl Med Mol Imaging (2020) 47(5):1147–57. doi: 10.1007/s00259-019-04615-x 31754795

[27] Seban RD Assie JB Giroux-Leprieur E Massiani MA Soussan M Bonardel G . FDG-PET biomarkers associated with long-term benefit from first-line immunotherapy in patients with advanced non-small cell lung cancer. Ann Nucl Med (2020) 34(12):968–74. doi: 10.1007/s12149-020-01539-7 33070295

[28] Vekens K Everaert H Neyns B Ilsen B Decoster L . The value of 18F-FDG PET/CT in predicting the response to PD-1 blocking immunotherapy in advanced NSCLC patients with high-level PD-L1 expression. Clin Lung Cancer (2021) 22(5):432–40. doi: 10.1016/j.cllc.2021.03.001 33879398

[29] Ferrari C Santo G Merenda N Branca A Mammucci P Pizzutilo P . Immune checkpoint inhibitors in advanced NSCLC: [18F]FDG PET/CT as a troubleshooter in treatment response. Diagnostics (Basel) (2021) 11(9):1681. doi: 10.3390/diagnostics11091681 34574022 PMC8471751

[30] Polverari G Ceci F Bertaglia V Reale ML Rampado O Gallio E . 18F-FDG pet parameters and radiomics features analysis in advanced nsclc treated with immunotherapy as predictors of therapy response and survival. Cancers (Basel) (2020) 12(5):1163. doi: 10.3390/cancers12051163 PMC7281558 32380754

[31] Mu W Tunali I Qi J Schabath MB Gillies RJ . Radiomics of 18F fluorodeoxyglucose PET/CT images predicts severe immune-related adverse events in patients with NSCLC. Radiol Artif Intell (2020) 2(1):e190063. doi: 10.1148/ryai.2019190063 33937811 PMC8074998

[32] Lopci E Toschi L Grizzi F Rahal D Olivari L Castino GF . Correlation of metabolic information on FDG-PET with tissue expression of immune markers in patients with non-small cell lung cancer (NSCLC) who are candidates for upfront surgery. Eur J Nucl Med Mol Imaging (2016) 43(11):1954–61. doi: 10.1007/s00259-016-3425-2 27251642

[33] Im HJ Bradshaw T Solaiyappan M Cho SY . Current methods to define metabolic tumor volume in positron emission tomography: Which one is better? Nucl Med Mol Imaging (2018) 52(1):5–15. doi: 10.1007/s13139-017-0493-6 29391907 PMC5777960

[34] Kim SI Cassella CR Byrne KT . Tumor burden and immunotherapy: Impact on immune infiltration and therapeutic outcomes. Front Immunol (2021) 11:629722. doi: 10.3389/fimmu.2020.629722 33597954 PMC7882695

[35] Guisier F Cousse S Jeanvoine M Thiberville L Salaun M . A rationale for surgical debulking to improve anti-PD1 therapy outcome in non small cell lung cancer. Sci Rep (2019) 9(1):16902. doi: 10.1038/s41598-019-52913-z 31729430 PMC6858444

[36] Wang Y Zhao N Wu Z Pan N Shen X Liu T . New insight on the correlation of metabolic status on 18F-FDG PET/CT with immune marker expression in patients with non-small cell lung cancer. Eur J Nucl Med Mol Imaging (2020) 47(5):1127–36. doi: 10.1007/s00259-019-04500-7 31502013

[37] Sumitomo R Hirai T Fujita M Murakami H Otake Y Huang CL . M2 tumor-associated macrophages promote tumor progression in non-small-cell lung cancer. Exp Ther Med (2019) 18(6):4490–8. doi: 10.3892/etm.2019.8068 PMC6862535 31777551

[38] Moon SH Hyun SH Choi JY . Prognostic significance of volume-based PET parameters in cancer patients. Korean J Radiol (2013) 14(1):1–12. doi: 10.3348/kjr.2013.14.1.1 23323025 PMC3542291

[39] Vaupel P Schmidberger H Mayer A . The warburg effect: Essential part of metabolic reprogramming and central contributor to cancer progression. Int J Radiat Biol (2019) 95(7):912–9. doi: 10.1080/09553002.2019.1589653 30822194

[40] Chang CH Qiu J O'Sullivan D Buck MD Noguchi T Curtis JD . Metabolic competition in the tumor microenvironment is a driver of cancer progression. Cell (2015) 162(6):1229–41. doi: 10.1016/j.cell.2015.08.016 PMC4864363 26321679

[41] Chang CH Curtis JD Maggi LB Jr Faubert B Villarino AV O'Sullivan D . Posttranscriptional control of T cell effector function by aerobic glycolysis. Cell (2013) 153(6):1239–51. doi: 10.1016/j.cell.2013.05.016 PMC3804311 23746840

[42] Harel M Ortenberg R Varanasi SK Mangalhara KC Mardamshina M Markovits E . Proteomics of melanoma response to immunotherapy reveals mitochondrial dependence. Cell (2019) 179(1):236–250.e18. doi: 10.1016/j.cell.2019.08.012 31495571 PMC7993352

[43] Fischer K Hoffmann P Voelkl S Meidenbauer N Ammer J Edinger M . Inhibitory effect of tumor cell-derived lactic acid on human T cells. Blood (2007) 109(9):3812–9. doi: 10.1182/blood-2006-07-035972 17255361

[44] Bosticardo M Ariotti S Losana G Bernabei P Forni G Novelli F . Biased activation of human T lymphocytes due to low extracellular pH is antagonized by B7/CD28 costimulation. Eur J Immunol (2001) 31(9):2829–38. doi: 10.1002/1521-4141(200109)31:9<2829::aid-immu2829>3.0.co;2-u 11536182

[45] Yıldırım F Yurdakul AS Özkaya S Akdemir ÜÖ Öztürk C . Total lesion glycolysis by 18F-FDG PET/CT is independent prognostic factor in patients with advanced non-small cell lung cancer. Clin Respir J (2017) 11(5):602–11. doi: 10.1111/crj.12391 26434685

[46] Zhang S Zhang R Gong W Wang C Zeng C Zhai Y . Positron emission tomography-computed tomography parameters predict efficacy of immunotherapy in head and neck squamous cell carcinomas. Front Oncol (2021) 11:728040. doi: 10.3389/fonc.2021.728040 34650916 PMC8506113

[47] Ayati N Sadeghi R Kiamanesh Z Lee ST Zakavi SR Scott AM . The value of 18F-FDG PET/CT for predicting or monitoring immunotherapy response in patients with metastatic melanoma: A systematic review and meta-analysis. Eur J Nucl Med Mol Imaging (2021) 48(2):428–48. doi: 10.1007/s00259-020-04967-9 32728798

